# Functionalization and Characterization of Silicon Nanowires for Sensing Applications: A Review

**DOI:** 10.3390/nano11040999

**Published:** 2021-04-13

**Authors:** Samuel Ahoulou, Etienne Perret, Jean-Marie Nedelec

**Affiliations:** 1 Université Clermont Auvergne, Clermont Auvergne INP, CNRS, ICCF, F-63000 Clermont-Ferrand, France; 2 LCIS, INP, University of Grenoble Alpes, Grenoble, F-26000 Valence, France; etienne.perret@lcis.grenoble-inp.fr

**Keywords:** silicon nanowires, functionalization, biological, gas, humidity sensors

## Abstract

Silicon nanowires are attractive materials from the point of view of their electrical properties or high surface-to-volume ratio, which makes them interesting for sensing applications. However, they can achieve a better performance by adjusting their surface properties with organic/inorganic compounds. This review gives an overview of the main techniques used to modify silicon nanowire surfaces as well as characterization techniques. A comparison was performed with the functionalization method developed, and some applications of modified silicon nanowires and their advantages on those non-modified are subsequently presented. In the final words, the future opportunities of functionalized silicon nanowires for chipless tag radio frequency identification (RFID) have been depicted.

## 1. Introduction

The first synthesis of silicon nanowires (SiNWs) was reported in 1957 by Treuting and Arnold [1]. At the time, these compounds were known as whiskers. However, work on silicon nanowires really started after the publication of Wagner and Ellis bearing on the description of the growth process of these nanowires using gaseous silane (SiH4) as a precursor and gold nanoparticles as catalysts [2]. The authors claimed that the addition of certain impurities on the surface of the silicon substrate are essential for the growth of silicon nanowires. Moreover, the silicon nanowires synthesized by this method are characterized by a small droplet of these impurities located at their tips. Known as the vapour-liquid-solid (VLS) mechanism, it represents a commonly-used way to synthesize silicon nanowires. Nevertheless, silicon nanowires can be synthesized by various top-down approaches such as e-beam [3], and especially by lithography [4,5]. The main disadvantage of these methods is their high cost. Bottom-up approaches are usually used to design low-cost SiNW devices. In this approach, the growth of nanowires takes place in gaseous environments. As examples we have chemical vapour deposition (CVD) [6,7,8], molecular beam epitaxy [9] (MBE), and low-pressure chemical vapour deposition [10,11,12] (LPCVD). We can also identify the solution-based growth techniques [13,14,15], suitable for high-yield SiNW production. In the mid-1990s, researchers found that these could be of potential interest for the electronics industry. Therefore, a huge amount research was dedicated to the study of their properties, notably their large surface-to-volume ratio, allowing a good interaction (molecule exchange or captions) at their surface, thus promoting their sensitivity. They can undergo n or p conduction changes in transport characteristics upon exposure to environments of varying pH. Exposed to the open air, a native silicon oxide (negatively charged at pH 3.5) is formed on the surface and acts as an ion-selective sensor [16]. 

Silicon nanowires have already been used as sensors in various fields, such as biological [17,18,19], chemical [16,20], and gas [21], but they suffer sometimes from low selectivity. An efficient sensor requires a high response, fast response time, high selectivity, chemical and physical stability, and low cost. However, the reactivity of organic compounds may allow to modulate the molecular structure of the sensing materials to enhance their selectivity towards a target analyte. Hence, the functionalization of organic layers on silicon surfaces could fill the lack of sensitivity and therefore lead to the manufacture of high-performance sensors, both in gaseous phase and liquid media. Furthermore, in the field of sensors, organic sensing materials such as conducting polymer can show a response to target analytes at room temperatures or low temperatures, which is very attractive in terms of operating conditions [22]. To the best of our knowledge, the first sensor based on organic molecules and biomolecules functionalized silicon wires both to illustrate protonation/deprotonation sensitivity and demonstrate selective bioactivity sensing date back to 2001 [23]. Following him, we note a handful of articles in the literature in 2003. Nevertheless, publications on silicon nanowires functionalization started to outnumber from 2005 to date. Only for 2020, we estimate at no less than 2000 the number of publications in the literature relating to the use of modified silicon nanowires as sensors. The purpose of this report is to present an overview of the different methods used for SiNW functionalization, especially with organic functions, as well as the different techniques used to characterize them. A comparative study between the different functionalization methods will be carried out to highlight the most suitable according to the targeted applications. At the end, we will show their applications as sensing materials. Special emphasis will be placed on some potential organic compounds likely to modify the silicon surface for the design humidity and gas sensors. 

## 2. Modification Methods and Their Characterization

The use of SiNWs for the specific detection of analytes requires that their surface has enough affinity with the compounds that we want to detect. Such a goal is generally achieved by modifying the surface properties with organic/inorganic components which promote the rise in the sensitivity of the materials. We distinguish several methods, both in solution and in the gas phase, allowing the functionalization of the silicon surface. In this part, we describe the main methods, namely silanization and hydrosilylation chemistry, but also provide an overview of other surface modification strategies explored in the literature, such as esterification, phosphorization, or layer by layer.

### 2.1. Esterification

Esterification reactions refer to a reaction between a carboxylic acid and alcohols which leads to the formation of an ester and water that can be expressed as:

(1) $$R-\mathrm{COOH}\;+\;R^{\prime }\;-\;\mathrm{OH}\to R\;-\;\mathrm{COO}\;-\;R^{\prime }\;+\;H_{2}O$$

**Scheme 1:**
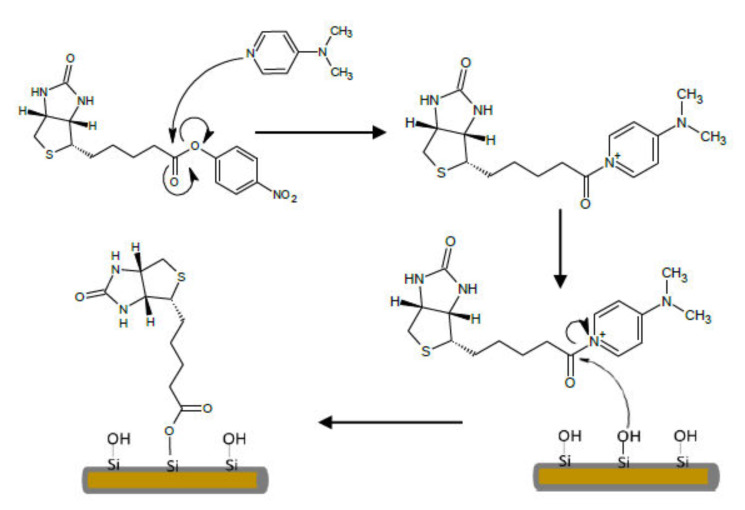
Mechanism of DMAP-mediated coupling between biotinyl p-nitrophenyl ester and the silanol groups of SiNWs.

This is typically a reversible process, widely used in many industrial applications like solvents for paints, pharmaceuticals, or food. Some ester derivatives can also act as a good intermediate for the bio-immobilization of high molecular weight polymers. Nonetheless, very few examples of esters modifying the silicon surface are reported in the literature, since the binding Si-O-CO is not sufficiently stable under hydrolysis compared to the Si-O-Si bond. Lieber et al. built a device based on silicon nanowires modified with peptide nucleic acid for the detection of DNA [24]. Firstly, the silicon surface was cleaned with oxygen plasma in order to remove any impurities and promote the production of hydroxyl groups. Then, SiNWs were soaked overnight at room temperature in pyridine solution containing biotinyl p-nitrophenyl ester and 4-(dimethylamino)pyridine (DMAP) which plays the role of the base. The esterification reaction is known to be a kinetically sluggish reaction, and thus DMAP serve also as catalyst since it can increase the velocity of the reaction up to 104 times, probably due to the stabilization of the acylpyridinium ion. The esterification reactions with carboxylic acid anhydrides in the presence of acidic catalysts are accompanied by chain degradation. The use of a tertiary base, commonly pyridinium or trimethylamine, is recommended to avoid this. The reaction begins by the activation of a biotin derivative by reacting with DMAP. An intermediary is then formed which reacts with the hydroxyl groups of the surfaces. The mechanism can be described as shown in Scheme 1.

### 2.2. Phosphorization

**Figure 1:**
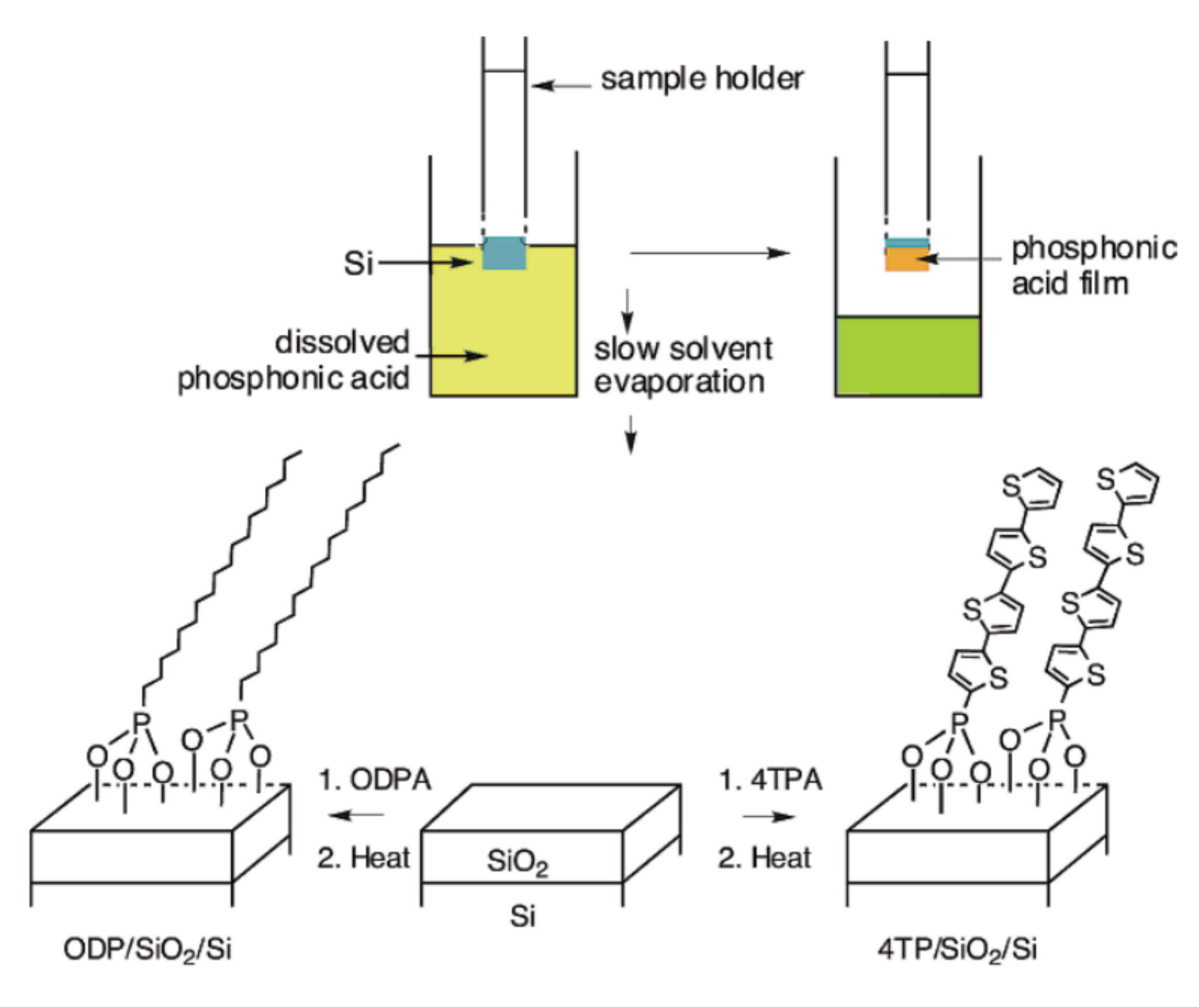
The T-BAG setup and chemical reactions. Reprinted with permission from [28], Eric L. Hanson et al., J. Am. Chem. Soc. 51, 16074–16080 (2003). © 2021, American Chemical Society.

Organophosphonates belong to a class of organophosphorus with general structure RPO_3_H_2_ (where R is an organic component) which can readily react with a wide range of metal salts and oxides. They are much less sensitive to nucleophilic substitution than the silicon parent derivatives and the bindings P-O-C are quite stable toward hydrolysis. The homocondensation of P-O-H bonds with the formation of P-O-P bonds takes place only under high-temperature dehydrating conditions. Therefore, the hybrid materials formed are monolayers, and show excellent properties. In addition, these have the advantage of being densely packed with higher surface coverage, great robustness, stable either in acidic or alkaline solutions, exhibiting excellent thermal stability and suitable for proton conductivity, especially at operating temperatures above 100 °C and in dry conditions [25]. Surface-modifying organophosphonates are generally acids and can be deposited on different variety of substrates, such as Al_2_O_3_, pure Ti, Ti-6Al-4V, and SiO_2_/Si [26,27]. Pioneered by Hanson et al. [28], the deposition method known as tethering by aggregation and growth (T-BAG) is based on the self-assembly of arranged amphiphilic molecules at the liquid–gas interface with the substrate. They reported the self-assembly of octadecylphosphonate and α-Quarterthiophene-2-phosphonate on oxidized silicon substrate. After the cleaning step, the substrate was held vertically using a small clamp in THF solution containing the organophosphonic acids below their critical micelle concentration. The reaction was performed at room temperature for 3 h to allow simultaneously the slow evaporation of the solvent and the gradual transfer of organophosphonic acid onto the Si surface until the level of solution dropped below the Si sample. Afterwards, the substrate was heated at 140 °C to bond the layer to the substrate (Figure 1). Once developed, the infrared spectrum of the modified silicon exhibited vibration bands attributed to an ordered alkyl-chain [29] linked to phosphonate. AFM images showed surface roughness in good agreement with the formation of a monolayer. Furthermore, quartz crystal microbalance (QCM) (an efficient technique to characterize the surface loading formed on SiO2/Si [26]) gave a value of 0.90 nmol/cm^2^ for octadecylphosphonate, consistent with close-packed alkyl chains.

Shang et al. [30] used almost the same approach to modified silicon microring resonator biosensor with 11-hydroxyundecylphosphonic acid. The presence of phosphonate groups was evinced by X-ray photoelectron spectroscopy (XPS) instrument from Kratos Analytical Ltd. (Manchester, UK), which gave a relative percentage of atoms corresponding to one-cycle self-assembly deposition. These attachments were further confirmed by time-of-flight secondary ion mass spectrometry (ToF-SIMS, from IONTOF GmbH, Münster, Germany) data, which clearly distinguished peaks of organophosphate fragments (PO_2_^−^, PO_3_^−^) from SiO_2_^−^. It should be noted that the heating of organophosphonic acid-adsorbed onto silicon substrates is mandatory for the formation of covalent bonds [31]. The authors claimed that the phosphonate, covalently attached, form a tridentate bond with silicon substrate and that one-cycle deposition may not be sufficient for 100% surface coverage. Indeed, we can identify three bonding modes of the organophosphonate head group with the surface oxide: (a) mono-dentate; (b) bi-dentate; and (c) tri-dentate [27,31]. The mechanism involves the condensation of -OH groups of phosphonic acids with the oxide layer on the metal substrate to form stable P-O-M linkages. In this process, surface OH groups may act as initial bonding sites, but the mechanism does not depend on them. High surface coverage is therefore not limited by surface OH content. As proof, Thissen et al. bonded monodentate organophosphate on a H-terminated Si(111) surface [32]. Moreover, Auernhammer et al. [33] modified directly a hydroxyl-terminated silicon carbide (6H-SiC) surface by 11-hydroxyundecyphosphonic acid (1) and 9,10-diphenyl-2,6-diphosphonoanthracene (2). The cleaning and hydroxylated steps were achieved by treatment with hydrofuran (HF) before functionalization. They highlight that two or three T-BAG sequences were necessary to obtain complete coverage of the semiconductor surface, respectively modified by (1) and (2). Between each sequence a careful washing was also performed to avoid multilayer formation due to hydrogen bonding among the phosphonic acid head-groups and hydroxyl group tails. As a characterization method, XPS, Fourier Transform Infrared Spectroscopy (FTIR) Analysis, and Quartz Crystal Microbalance (QCM) were used to evidence the modification. For more information about the properties and applications of organophosphonate monolayers, readers can refer to these papers [34,35]. Despite their interesting properties, phosphonic-acid-functionalized inorganic surfaces present some drawbacks, especially the low packing densities obtained for bulky organic groups attached to the phosphonic acid.

**Figure 2:**
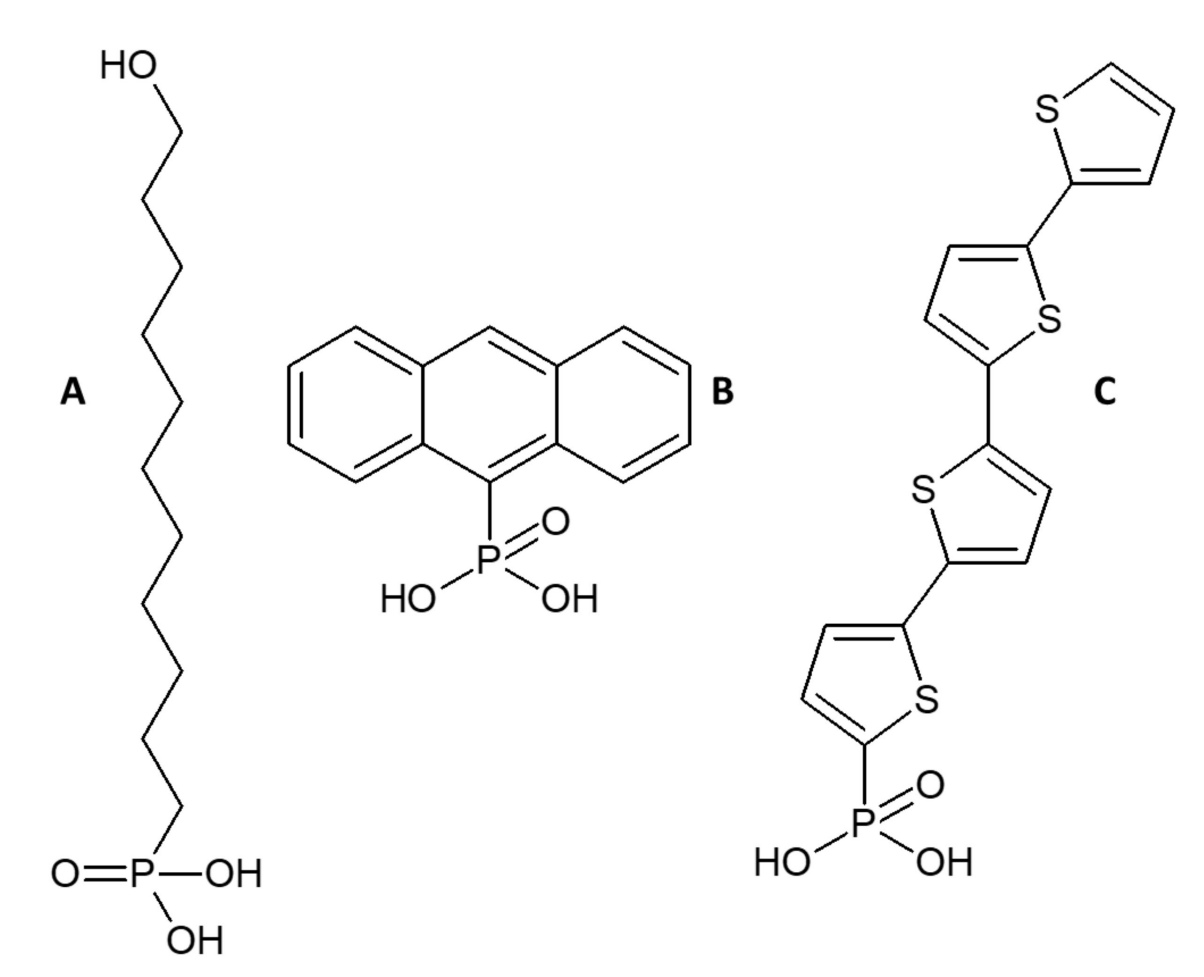
(**A**) 11-hydroxyundecylphosphonic acid; (**B**) (9-phosphono) anthracene; (**C**) α-quarterthiophene-2-phosphonates.

Additionally, Silicon surface modification by phosphorus can take place in the gas phase. Taghinejad et al. used bubbling POCl_3_ as a source for phosphorus diffusion to a modified SiNW surface by post-growth doping. The process was performed in a quartz diffusion furnace with a temperature set to 700 °C to favour the diffusion of POCl_3_ [10]. EDX measurements performed after doping showed the appearance of peaks at 2 KeV, which were attributed to the presence of phosphorus. Indeed, this peak did not appear before doping, thus highlighting the success of the doping process. Garipcan et al. investigated the in vitro biocompatibility of undoped and phosphorus-doped SiNWs, using phosphine (PH_3_) [36]. They characterized them by scanning electron microscopy, then studied their electronic properties. In principle, post-doping schemes should provide better control of concentration and afford the option of spatial selectivity. Nonetheless, modified SiNWs were also produced in one step by the simultaneous introduction of additional gases during the growth process [37,38]. Wang et al. investigated the efficiency of phosphine (PH_3_) as a gas dopant during Au-catalysed SiNW growth using the VLS method while varying the phosphorus-to-silicon ratio in the inlet gas stream [39]. The structural features of doped SiNWs highlighted by TEM exhibited a slight increase in surface roughness compared to nominally undoped samples. The concentration of phosphorus incorporated into the SiNWs was then determined by ion mass spectrometry (SIMS). Figure 2 shows some examples of organophosphonate compounds used to modify silicon surface.

### 2.3. Hydrosilylation

As mentioned above, SiNW surfaces are covered by native oxide SiOx. However, this can have some drawbacks in terms of performance. For example, monolayers formed on SiOx have low chemical stability in aqueous media due to the hydrolysis of Si-O bonds and this can result to their destruction [40]. Native oxides are a contamination source of oxygen and metallic impurities. Moreover, the functionalization of oxide-free SiNWs is considered to improve the sensing effect of SiNW devices because of the vicinity of targets/analyte interactions [17]. Hydrosilylation consists of inserting unsaturated bonds (alkynes or alkenes) into a silicon-hydride group to form a Si-C bond. This process involves the etching of the silicon oxide layer, commonly performed using a wet chemical approach, and the saturation of exposed surface bonds.

Wet chemical reactions require a metastable surface to successfully carry out surface chemistry. The precursor surface must be:

- Stable enough to handle at atmospheric pressure in the presence of solvent vapours.
- Inert to gas impurities and other contaminants.
- Reactive enough to undergo chemistry.

Note that a H-terminated silicon surface is relatively stable in air for short periods. In practical terms, hydrogen-terminated surfaces were obtained using fluoride-containing solutions, with dilute (<10%) hydrofluoric acid (to passivate the Si(100) orientation) and 40% ammonium fluoride solutions (for Si(111) face passivation) [41,42]. Indeed, the Si-F bond is highly polarized in the direction ^δ+^Si-F^δ−^, which enables simple substitution at the Si center upon nucleophilic attack and hence is kinetically very labile. This results in the formation of a surface that is mainly occupied by SiH_2_ and Si-H groups, respectively, for Si(100) and Si(111). Other chemical etchants, such as aqueous sodium hydroxide [43], can be used. Furthermore, ultra-high vacuum (UHV) techniques are used for the elimination of surface oxide layers [44]. H-terminated silicon surface reactions with alkenes or alkynes to form a Si-C bonded monolayer begin necessarily with the generation of Si radicals by breaking Si-H bonds [45]. Infrared spectroscopy can help to highlight Si-H formation. It is possible to obtain the latter using high temperatures, UV and visible light irradiation, or electrochemistry.

#### 2.3.1. Thermally-Induced Hydrosilylation of Unsaturated Molecules

**Figure 3:**
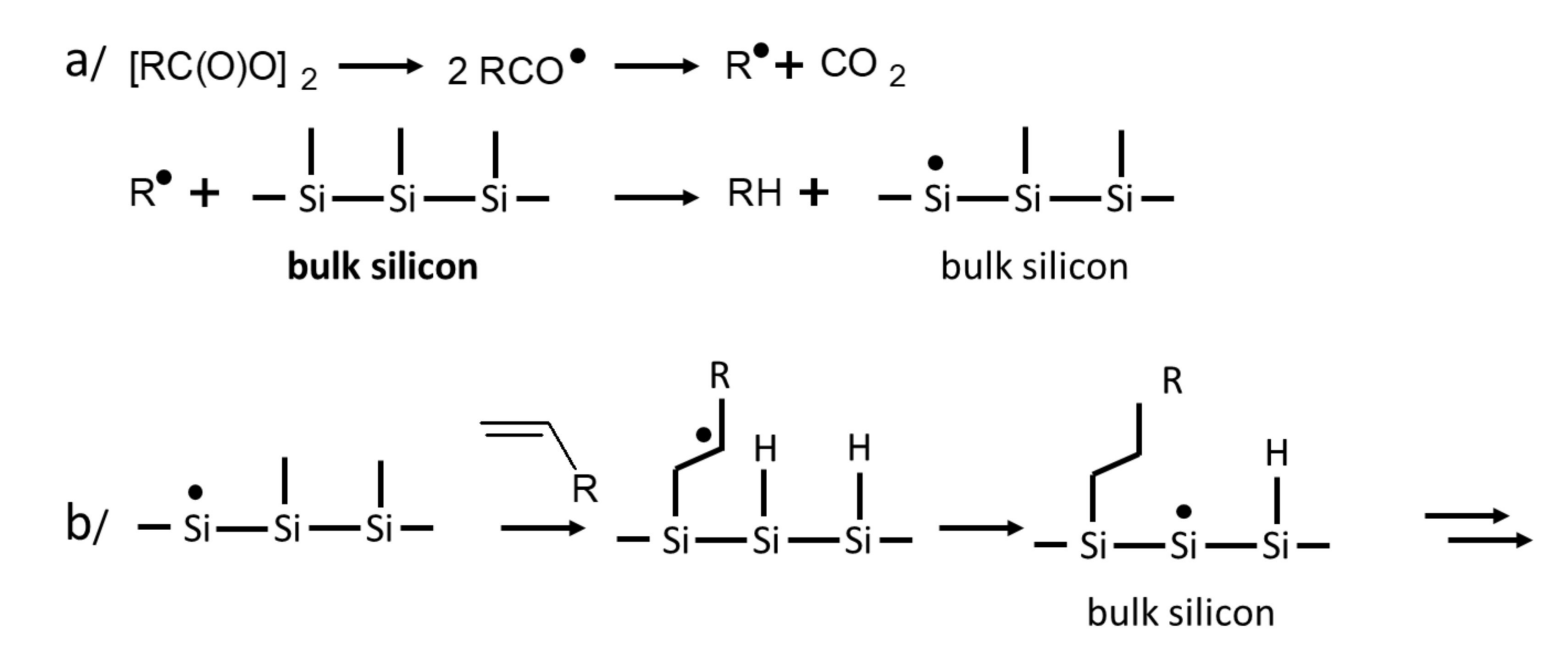
Mechanism for radical-based hydrosilylation, (**a**) Silicon radical formation process, (**b**) Reaction of alkene molecules with silicon radical.

The first example of hydrosilylation was reported by Linford and Chidsey in 1993 [46]. The functionalization of alkenes into surface-bound Si-H groups formed high-quality and densely-packed octadecyl monolayers in the presence of diacyl peroxide as a radical initiator at 100 °C. The silicon surface exhibited excellent stability in solution (water, chloroform, acid, or basic solution) even if a small oxidation was observed when exposed to air under ambient conditions. The authors suggested that alkyl monolayer formation was based on a radical mechanism initiated by diacyl peroxide. This latter undergoes homolytic cleavage to form two acyloxy radicals which decompose to carbon dioxide and an alkyl radical. Then the alkyl radical abstracts H^•^ from a surface Si-H group to produce a silicon radical (Si^•^) (Figure 3a) which can react with alkene to give a carbon-centred radical on the grafted molecule. This latter, in turn, can abstract hydrogen atoms at an adjacent Si-H site to give a new silicon radical and thus promote a new alkene addition site (Figure 3b) [45]. However, further investigations by Chidsey et al. demonstrated that hydrosilylation could occur at higher temperatures (≥150 °C) without diacyl peroxide.

This leads to a silicon radical which later reacts with olefine to form alkyl monolayers, stable up to 615 K under vacuum. Bateman et al. modified porous silicon by direct reaction with 1-octene, 1-octyne and 1-undecene. These samples were dissolved in toluene and heated at reflux (110–180 °C) for 18–20 h. The reactions monitored by FT-IR spectroscopy, before and after functionalization, exhibited the presence of bands at 2900 cm^−1^, 1465 cm^−1^ and 1380 cm^−1^ typical of C-H bonds, which was not observed for hydride-passivated silicon. Ferrocenyl-derivatized porous silicon was also prepared in the same way and characterized by cyclic voltammetry [47]. Feng and co-workers also found that thermally-driven chemical reactions occurred between C60 monolayer molecules and H-terminated Si(100) substrate surfaces. After treatment to form a Si-H surface, the wafer reacted with C60 to form the C60 monolayer, either by solvent casting under Ar flow at 180 °C to 210 °C for 1.5 h or by vapour deposition in a quartz tube at 190–230 °C [48]. In 2000, Sieval et al. presented the first investigation of the thermal reaction of 1-alkynes of various lengths with a H-terminated Si(100) surface. Monolayer formation was characterized by water contact angle measurements, attenuated total reflection (ATR) infrared spectroscopy, and X-ray reflectivity. From the water contact angle data, the authors deduced ordered monolayers on the Si surface comparable to the values reported for thiols on gold [49]. In addition, the infrared spectroscopy spectra showed bands in the C-H stretching region [50]. The main limitation of the thermal hydrosilylation approach is the large excess of unsaturated molecules required. Henriksson et al. reported a more sophisticated method through click chemistry. Non-oxidized silicon nanowires were heated at 120 °C for 12 h with 1,2-Ethanediol-dipropiolate (EDDP). The terminal alkyne groups of this monolayer reacted at 50 °C with 1-azidooctane under the formation of the triazole-bridged addition product. Infrared spectra reveal the disappearance of wavenumbers at 2121 cm^−1^, corresponding to alkyne groups, indicating alkyne-azide cycloaddition [51].

#### 2.3.2. UV and Visible Light Irradiation-induced Hydrosilylation of Unsaturated Molecules

Pioneered by Terry et al. in a study dedicated to the direct evidence of silicon-carbon bond formation for the reaction of 1-pentene with Si(111)-H [52], the UV-mediated hydrosilylation reaction of unsaturated hydrocarbons appears as the most applied approach for H-terminated SiNW functionalization. The process is generally easy to set up, and favours both chemical stability and good surface coverage. The argument of a homolytic dissociation of surface Si-H (and Si-Si) not being possible to sustain under white light illumination, the proposed mechanism would be light-induced radical formation from impurities in solution and a hole-related mechanism favouring the reaction on illuminated substrates [53,54]. Streifer et al. were the first to report the use of photochemical hydrosilylation to covalently functionalize silicon nanowires with DNA or other biomolecules in order to achieve biomolecular recognition [55]. The reaction starts by the covalent attachment of primary amines, protected by t-BOC, to SiNWs using ultraviolet light at a wavelength of 254 nm. After the deprotection step, these amine groups react with an intermediate cross-linker sulfo-succinimidyl 4-(N-maleimidomethyl) cyclohexane-1- carboxylate, which will subsequently react with the DNA. Surface characterization performed by XPS has proved that the photochemical attachment of the t-BOC-protected amine proceeds without any significant oxidation of the sample. This strategy was also followed by Zhang et al. to design biosensors based on silicon nanowires modified by PNA-DNA hybridization. From TEM characterization, the authors concluded that the surface was uniform and oxide free.

#### 2.3.3. Electrochemical Approaches to Si-C-bound Hydrocarbon Monolayers

**Scheme 2:**
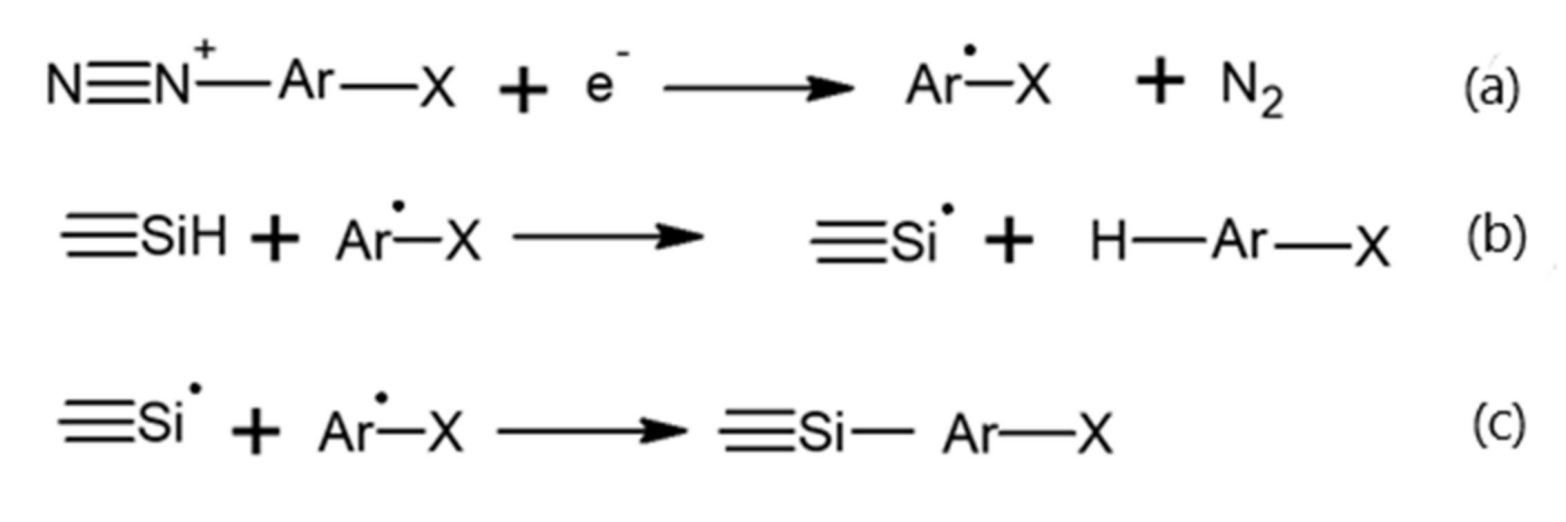
Mechanism of electrochemical reduction of diazonium salts on hydride silicon.

**Figure 4:**
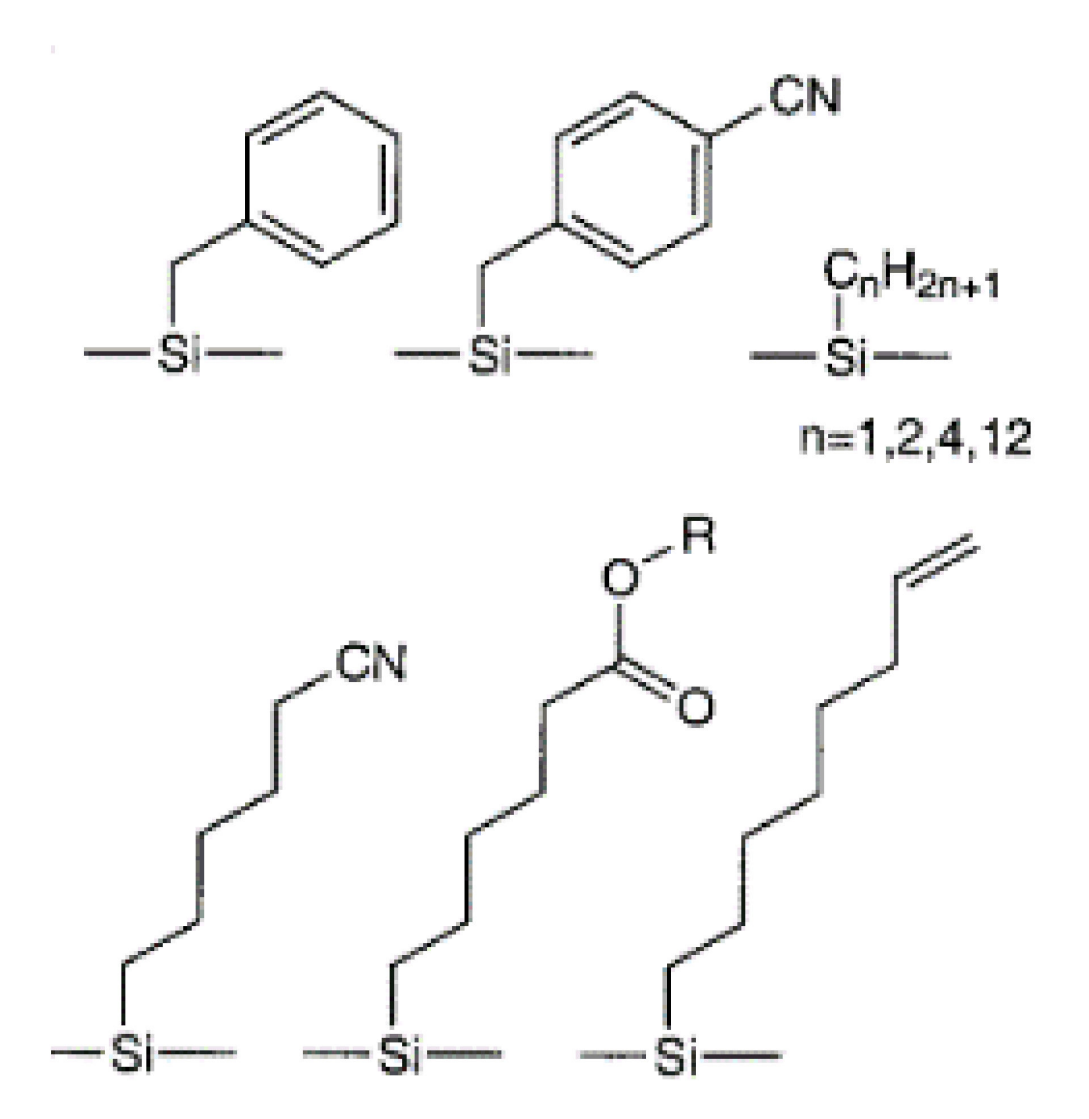
Examples of hydrosilylated surfaces modified by organic groups.

Electrochemistry was used by Allongue et al. [56,57] to produce phenyl layers on flat n-type Si(111) by the reduction of aryl diazonium salts (X-Ar-N2+; X = NO_2_, Br, COOH, CN). Under cathodic conditions of about 1V in diluted hydrofluoric acid (2%), diazonium salts are susceptible to produce dinitrogen and an aryl radical (Scheme 2a). The latter can then abstract a surface hydride to form silicon radicals (Scheme 2b), which can react with another aryl radical to form the silicon–carbon bond (Scheme 2c). The proposed mechanism of electrochemical reduction of diazonium salts on hydride silicon is summarized as follows [56]:

The covalent nature of prepared Si-C bound layers is confirmed by their stability in 40% HF solution, STM imaging, angle resolved XPS, capacitance measurements and Rutherford backscattering. This approach has the advantage of limiting silicon oxidation in the final surface because the cathodic process makes the surface rich in electrons during the reaction, and thus less susceptible to nucleophilic attack by water. Some examples of organic compounds modified by this method are shown in Figure 4.

**Figure 5:**
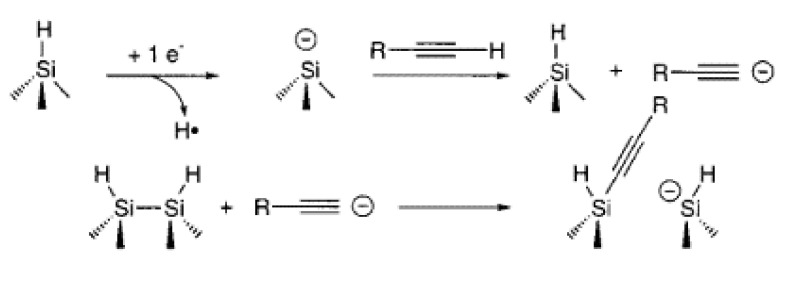
Reaction mechanism of silicon-bonded monolayers produced by electrografting.

Terminal alkynes can also form silicon-bonded monolayers by electrografting under a negative bias [58]. In this case, the silylated alkyne products start with the formation of silyl anions after the reduction of a Si-H site, followed by an acid/base reaction to give a carbanion, and the subsequent nucleophilic attack of a Si-Si bond (Figure 5). The surface-bonded alkyne was observed by infrared measurements with vibration around 2179 cm^−1^. Note that the reaction is more specific for acetylenes. The reaction scheme is described as follows:

Apart from the various hydrosilylation techniques describe above, Lewis acid (e.g., AlEtCl_2_, AlCl_3_) was used to mediate the hydrosilylation of alkynes and alkenes [59,60]. This allows a high selectivity and specificity of the corresponding solution-phase reaction. H atoms are the most commonly used passivating agent for surface preparation, but some approaches based on halogen-terminated surfaces have been reported [61]. Haick et al. explored the electrical properties and chemical stability of non-oxidized silicon nanowires modified by methyl groups. The modification was achieved in two steps via a chlorination/alkylation route. The first step was achieved by the immersion of H-SiNWs into a saturated solution of PCl5 dissolved in C_6_H_5_Cl. The reaction, triggered by diacyl peroxide, was heated to 90–100 °C. The chlorine terminated SiNWs were alkylated by the Grignard route [62].

#### 2.3.4. Silanization

**Figure 6:**
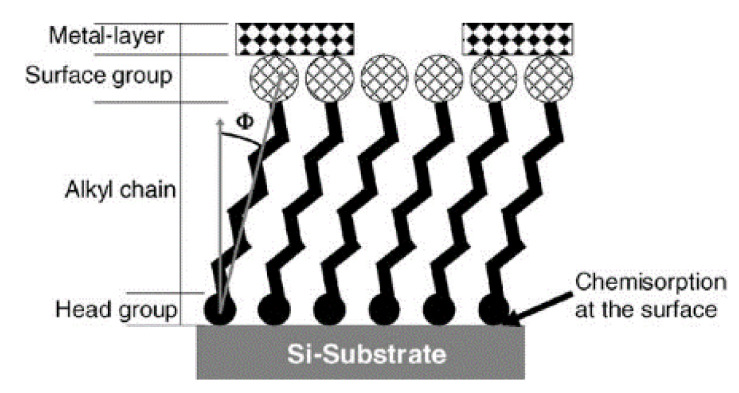
Schematic diagram showing the different surfactant molecules linking to the Si-substrate.

The silanization reaction is described as the binding of organofunctional groups to the oxide shell covering the silicon surface to form siloxane bonds (Si-O-Si) [63]. These surfactant molecules R(CH_2_) nSiX_3_ (X = Cl, OCH_3_ or OC_2_H_5_), self-assembled on the silica surface, can be divided into three parts. Firstly, we have the Head group (-SiX_3_) which forms the chemical bond with the substrate. A high density of these leads to a good surface coverage. Thereafter, we have an alkyl chain -(CH_2_)n- functioning as spacer groups, and finally the surface group (-R), which can be substituted with different functional groups according to the desired applications (Figure 6). 

**Figure 7:**
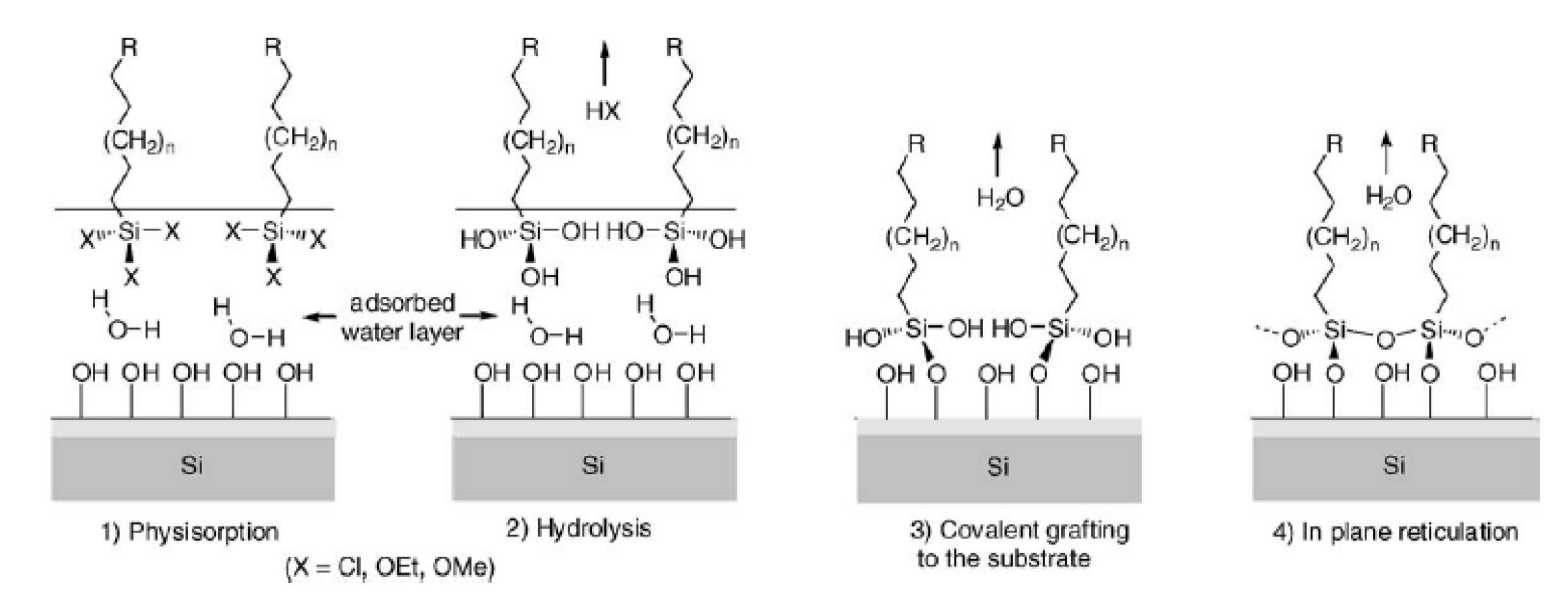
Silanization mechanism. Reprinted from The Lancet, 568, D.K. Aswal et al., Self-assembled monolayers on silicon. © (2006), with permission from Elsevier.

Silane compounds are commonly used on oxide surfaces, where they can covalently bind to the surface by the transfer of a proton from the hydroxylated surface to a silane leaving group, eliminating an alcohol (in the case of methoxy or ethoxysilanes) or HCl (in case of chlorosilanes) [64,65,66,67]. As a reminder, Langmuir and Blodgett’s (LB) technique, discovered in 1920, was the most often used to design organic monolayers on substrates. However, the layer formed was physisorbed, and therefore weaker [68]. The oxide layer formed on the silicon surface, at room temperature, contains a high density of traps, which are unfavourable for electrical purposes. For this reason, it is recommended to eliminate this layer and to thermally grow a new one. In all cases, deep cleaning of the substrate is a prerequisite to obtain a clean oxide layer with high density of silanol groups (Si-OH) on the surface, acting as anchor sites for silanization reactions. The cleaning step is usually performed using piranha solution (H_2_SO_4_/H_2_O_2_). In general, the functionalization is carried out by the simple immersion of the freshly hydroxylated surface into an organic solution containing a silane derivative [69,70]. Toluene is commonly used as a reaction medium. The SAM formation begins with the physisorption of silane molecules on the hydrated silicon surface. Next, the silane head-groups are hydrolysed (-SiX_3_ → -Si(OH)_3_; X = Cl, OEt, OMe) in the presence of the water layer at the substrate surface, and then they form covalent bonds with the hydroxyl groups on the SiO_2_ surface (Figure 7). It should also be noted that silanol groups attached to the silicon surface are susceptible to condensing with these neighbours [71,72].

**Figure 8:**
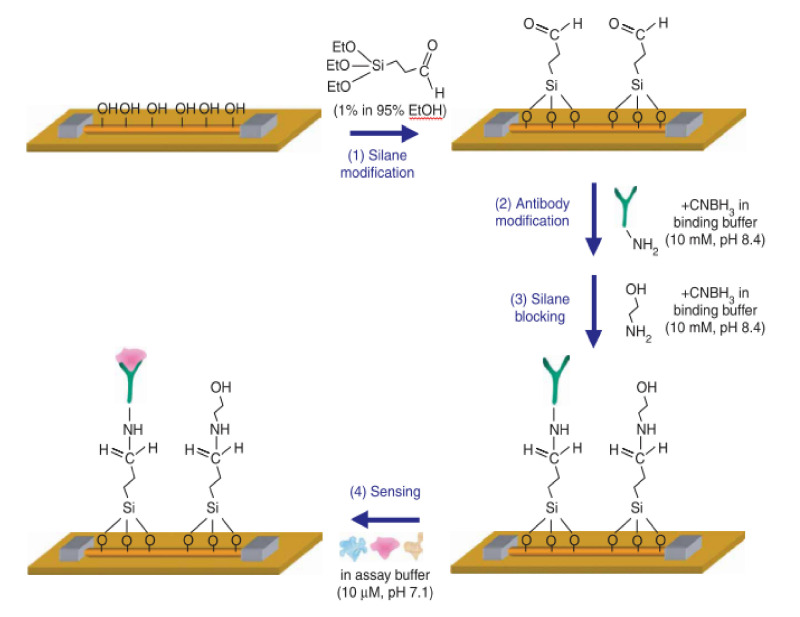
Silicon nanowire modification scheme for DNA recognition [74].

As a selective sensor can generally be configured from silicon nanowire devices by linking recognition receptor groups to the surface of the nanowire [73], silanization remains the most frequently-applied method to functionalize the native silicon oxide coating on silicon nanowires. Patolsky et al. constructed a biological and chemical species sensor by linking prostate-specific antigen (PSA) monoclonal antibody (receptor) to the SiNW surface. After the cleaning step with oxygen plasma, functionalization was achieved by the immersion of the sensor chip for 30 min in a silane ethanol/solution containing 3-(trimethoxysilyl)propyl aldehyde. This latter was subsequently washed with ethanol and heated at 110 °C before being submerged in the antibody solution. With a view to preventing nonspecific adsorption of the target species, the authors passivated unreacted aldehyde groups on the silicon surface using an ethanolamine solution. To demonstrate the surface modification procedures, electrical measurements were carried out [74] (Figure 8).

Salhi et al. also reported silicon modification, on one hand by alkyl chain and on the other hand by sulfonate groups [75]. Firstly, the silicon surfaces were cleaned in a piranha solution (3:1 concentrated H_2_SO_4_/30% H_2_O_2_) at 80 °C. The alkyl-modified silicon surface was produced by soaking the hydroxyl surface in hexane solution with 10-3 M octadecyltrimethoxysilane (OTS) for 2 h at room temperature under a nitrogen atmosphere. Surface functionalization of the SiNWs by sulfonate groups was performed in two steps. The first was to shape the thiol layer on the silicon surface by submerging it in 3-mercaptopropyltrimethoxysilane dissolved in toluene. Subsequently, the oxidation of the thiol groups to the desired sulfonate groups was carried out by dipping the substrates in a nitric acid aqueous solution (30%). The success of the oxidation reaction was confirmed by XPS analyses. Apart from the examples developed above, the silanization method has been applied to different functional groups: -COOH, -CN [76]; -NH_2_ [77,78,79,80]; -Br, -COOCH_3_ [81]; alkyl [82,83]. We should point out that a long hydrocarbon chain tends to reduce thermal stability and change the mechanical properties. Likewise, the alkyl surface concentration decreases as the n-alkyl chain length increases [84]. Let us focus for a moment on 3-aminopropyltriethoxysilane (APTES), an alkoxysilane which is a commonly used to modify silicon nanowires and is the main coupling agent to synthesize aminated silane films. Indeed, APTES favours the adhesions of polymer films on glass [85], promotes protein adhesion [86] for biological implants, and is used to attach metal nanoparticles to silica substrates [87]. However, APTES films are subject to forming disordered monolayers or multilayers because of the favourable head-and-tail group interactions [88,89]. Even though the problem of disordered monolayers can be overcome by using an intense electrical field [90], it is necessary to establish a set of conditions favouring good film quality. Howarter and Youngblood investigated the optimal conditions for solvent-based silanization using APTES [80]. The authors highlighted the fact that the morphology and groth kinetics of APTES films were affected by reaction time, solution concentration, and temperature. Experiments with an APTES concentration of 1% only produced acceptable films when the reaction time was restricted to 1 h. Increasing the reaction time increased the APTES film thickness. Suspène et al. carried out an interesting comparative study on triphenylamine grafted onto the silicon surface both by hydrosilylation and silanization [91]. They showed that silanization enabled a higher density of organic compounds around SiNWs and thus maximized the intensity of spectra recorded by spectroscopic techniques. The modified SiNWs were characterized by UV-visible, XPS and cyclic voltammetry. 

**Figure 9:**
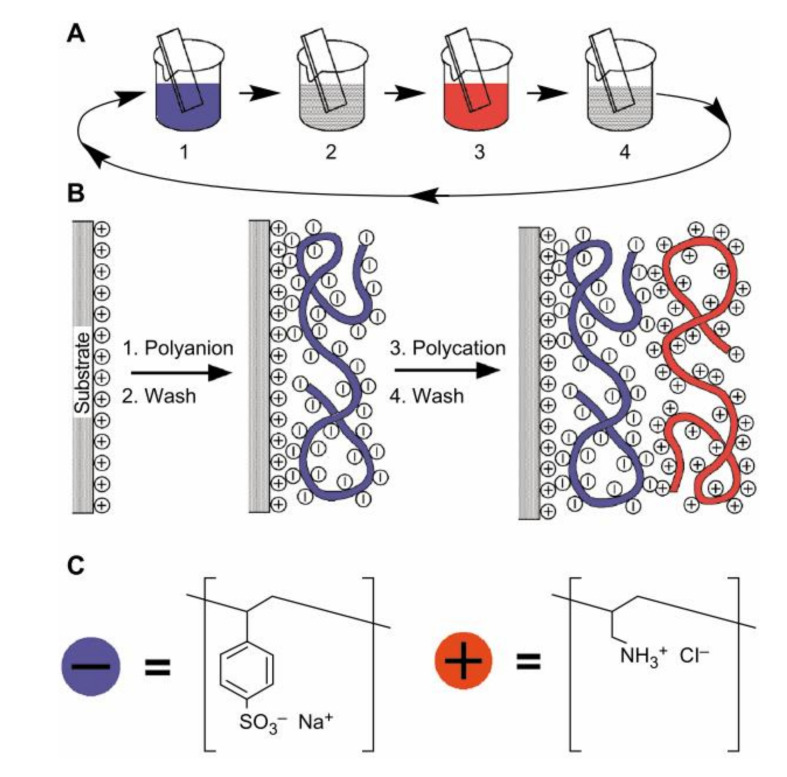
(**a**) Schematic process of film deposition on a flat substrate. Steps 1 and 3 correspond respectively to the adsorption of the polyanion and to the polycation while steps 2 and 4 are washing steps; (**b**) simplified image of the film formed on the substrate during steps 1 and 3; (**c**) chemical structures of the polyions used: sodium salt of poly(styrene sulfonate) (violet) and poly(allylamine hydrochloride) [92].

There exists an alternative approach to modifying the silicon oxide layer by electrostatic interaction. Known as layer-by-layer (LbL) deposition [92], the process takes place in solution and involves the successive adsorption of oppositely-charged species on the substrate surface, as schematically outlined in Figure 9. Film deposition can be carried out by different techniques, including dip-coating, spin-coating, spray-coating. Adsorption times per layer range from seconds to minutes [76,92]. Film deposition is generally achieved using an adsorbate concentrations of several milligrams per millilitre to avoid impoverishment of the solution during the fabrication of the multilayer stack. Note that the film thickness increases linearly with the number of deposition cycles. Polyelectrolytes (polyanion or polycation) are very often preferred to small molecules because of their strong electrostatic attraction, favouring the good adhesion of the layer to the underlying substrate. However, the properties of the final materials are closer to those of the polymer than those of underlying substrate. Vu et al. designed a functionalized silicon nanowire biosensor chip by the LbL adsorption of polyelectrolytes using dip coating. First, polyallylamine hydrochloride (PAH: MW 15,000) was immobilized on oxidized silicon. Then, they added polystyrenesulphonate (PSS: MW 60,000–80,000). This sequence was repeated several times to build a stack of six bilayers [93].

**Figure 10:**
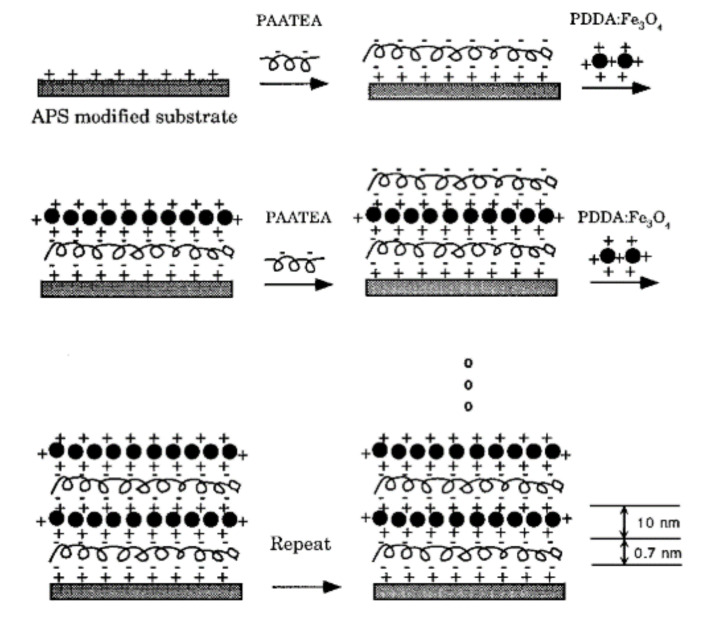
Illustration of multilayer formation by electrostatic molecular self-assembly. Reprinted from Liu, Wang, and Claus, Appl. Phys. Lett., Vol. 71, 1997 with the permission of AIP Publishing.

Liu et al. reported the synthesis of metal–organic frameworks (MOFs) on surface-modified SiNWs [76]. The functionalization process started by the formation of -CN groups at the silicon oxide surface, which were hydrolyzed to form -COOH-modified SiNWs. The nanowires obtained were successively soaked in 10 mmol/L copper acetate (Cu(OAc)_2_ ethanol solution and 50 mmol/L H_3_btc (btc = 1,3,5-benzenetricarboxylate) ethanol solution at room temperature for 10, 20, or 40 cycles. The sample was washed after each soaking with ethanol to remove excess reactant from the surface. The characterization of MOFs on SiNWs was performed by XPS, X-ray diffraction, scanning electron microscopy and transmission electron microscopy. The authors indicated that the thickness of the MOF shell was about 80–120 nm after 40 cycles, implying an average thickness of 2–3 nm for each deposition cycle. Claus et al. also showed the preparation of monolayer and multilayer magnetic Fe_3_O_4_/polyimide films on single silicon crystal [94]. As before, several steps are involved in the fabrication process. The substrates were first modified by (N-2-aminoethyl-3-aminopropyl) trimethoxysilane) via silanization. A positive charge was applied to the amine monolayer by adjusting the pH and immersion in an aqueous solution of polyimide precursor at pH 8.5 for 10 min. Subsequently, the slide was dipped into a mixture of cationic solution (Fe_3_O_4_/poly(diallyldimethylammonium chloride) with a pH of 8.5 for 10 min. By the repetition of these two processes in a cyclic fashion, a multilayer film was obtained, in principle without any limitation on the thickness of the final materials (Figure 10). The major advantages of layer-by-layer adsorption from solution are that many different materials can be incorporated into individual multilayer films, and the film architectures are completely determined by the deposition sequence.

The Table 1 summarize the main characteristics of each functionalization approach. For nanosized materials, surface properties are become paramount due to their large surface-to-buck ratio. This makes them particularly attracting in the applications where such properties are exploited. On the grounds that silicon surface can be facilely functionalized, leading in this way to different surface properties (high surface-to-volume ratio favourable for molecule adsorption, efficient conduction, mechanical and optical properties), the chemical sensitivity on silicon nanowires has been exploited to design chemical biosensors and sensors [16,20,95,96]. 

## 3. Sensing Properties of Modified Silicon Nanowires

**Figure 11:**
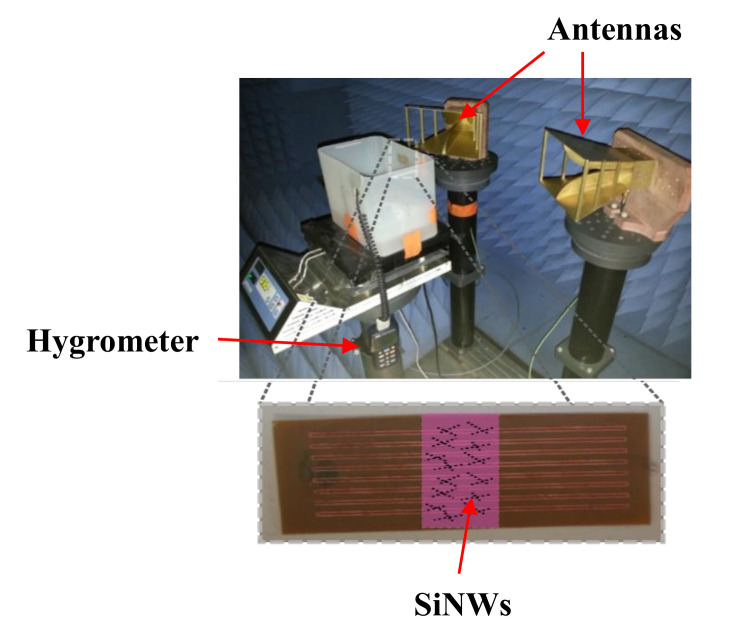
Radio frequency measurements set up for hygrometry variation [95].

The principle of sensors (defined as devices that incorporated with sensing materials) detection is based on interaction between a molecular probe and its target, thus the change of physical properties of the sensing material is translated into a quantifiable signal via a transducer. In terms of applications, RFID technology is an example that exploits this principle to transform RFID tags into sensors. Indeed, the radio frequency identification (RFID) is one of the major technologies in the field of identification and has grown considerably, since its principle was introduced 60 years ago [97]. This is a technique for the automatic capture of information contained in a label, by radio waves with a remote reading facility. Even if the information exchanged is mainly for identification purposes, more and more tags are used as wireless sensors [98]. However, the RFID solution is complex; it requires the use of a chip and a communication protocol which induces costly tags. It is why new identification and sensor systems are expected. Among them, a solution without any chip is very promising [98]. This is why the development of chipless radar tags in RF has grown significantly in recent years [98,99]. The principle of information encoding is based on the generation of a specific electromagnetic signature, on the basis of a radar principle (a wave is sent to the tag, and the significant information is contained in the tag backscattered signal). The shape of the conductive pattern forming the tag is imposed in order to have a specific and perfectly recognizable signature. Apart from that, the appearance of the tag is similar to that of a barcode. Thus, the information is no longer stored with an electronic chip, as can be done in traditional RFID tags, but directly "written" on the label. Therefore, a key point is the relationship between the geometry of the conductor pattern and the RF signature expected. Most often these shapes define resonators whose resonance frequencies are in the UWB band (between 3.1 and 10 GHz) [100,101]. A significant number of papers have now been published that explore the use of silicon nanowires in sensor devices. Many of them are based on the reading principle used in chipless RFID. An example, RFID humidity sensors were realized by deposing few drops of SiNWs (dissolved in ethanol) in the center of the scatterer where the electric field is maximum [95]. A random distribution of nanowires was observed after alcohol evaporation. Briefly, RF measurement consists in irradiating a target with an electromagnetic field then measuring the backscattered field. The variation of electromagnetic response is related to the use of some sensitive materials which induce variable conductivity and permittivity. The change in conductivity will in turn lead to a variation in the tag response level, whereas the permittivity will impact the resonant frequency or the phase of the scatterer. The Figure 11 shows the experimental set-up made of two antennas in an anechoic chamber, a plastic box partially filled with water (in front of antennas) and a chipless tag modified with SiNWs placed inside the box. A probe immersed in the water and connected to a hygrometer is used to monitor changes in temperature and relative humidity (RH).

**Figure 12:**
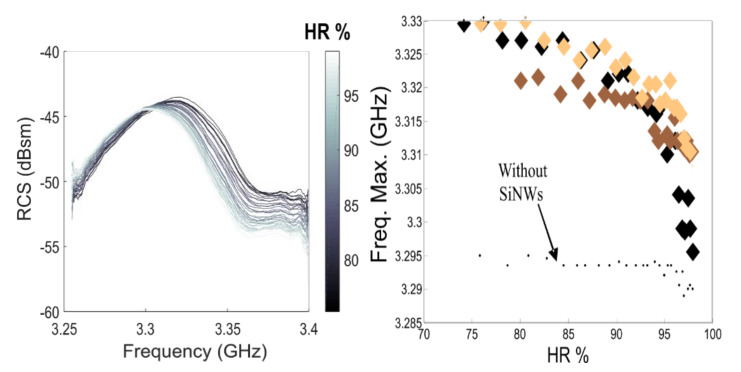
(**a**) Recorded frequency responses when RH rises from 74 to 98%; (**b**) Frequency shift as a function of the RH for the tag with and without SiNWs [95].

A frequency shift of 35 MHz was observed when the relative humidity increases from 74 to 98% (Figure 12a). The observed change was attributed to the strong sensitivity of the SiNWs to the humidity. The reproducibility of measurement was studied by comparing the variation in resonance frequency of four different measurements carried out on the same tag. They observed maximum error of 9.58% which decreases considerably at higher RH values. These results reveal that the sensors do not provide an absolute value but a threshold value. In order to verify the influence of silicon nanowires, the authors performed the same measurements on prototype without nanowires which shown no significant variation in the same humidity range (Figure 12b). This example provides proof of an extremely simple use of SiNWs for the realization of a wireless sensor. Therefore, using a radar approach with a label consisting solely of a conductive pattern and a localized deposit of SiNWs, it is possible to trace the humidity value in the air at a distance of a few tens of centimeters.

**Figure 13:**
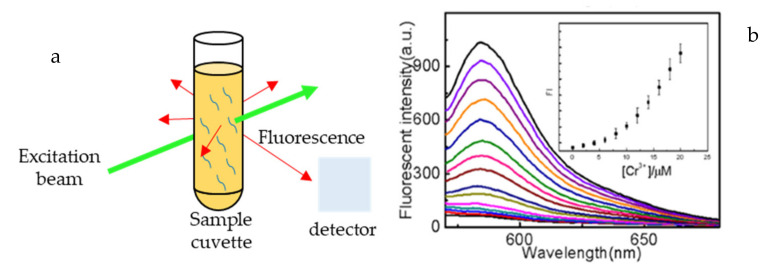
(**a**) Basic design of a fluorescence; (**b**) Fluorescence spectra and titration curves (Inset) of spirocyclic rhodamine modified SiNWs in presence of Cr^3+^.

Nevertheless, we notice that most silicon nanowires used in the manufacture of sensor devices requires a prior surface modification to control their stability, trap states, electrical properties, and physical/chemical interaction with analytes [102,103]. A silicon nanowire array was employed as a substrate to covalently graft various fluoroionophores for the detection of heavy metals ions [104] since it permits high carrier mobility, and so high sensitivity to analytes adsorbed on their surfaces. Fluorescence method was used to investigate the sensing effect due to its high sensitivity and simplicity for environmental analysis. First of all, suspensions of SiNWs-based fluoroionophores was dispersed in a mixture of ethanol and 2-[4-(2-hydroxyethyl) piperazin-1-yl] ethanesulfonic acid (HEPES) buffer at pH 7.4. Then, fluorescence spectra were recorded by means of beam with various wavelength (for Pb^2+^: λ_ex_ = 350–385 nm, for Cd^2+^: λ_ex_ = 470–495 nm, for Cr^3+^: λ_ex_ = 530–550 nm, for Hg^2+^: λ_ex_= 530–550 nm), crossing the solution upon the addition of different concentrations of heavy metals (Figure 13a).

**Figure 14:**
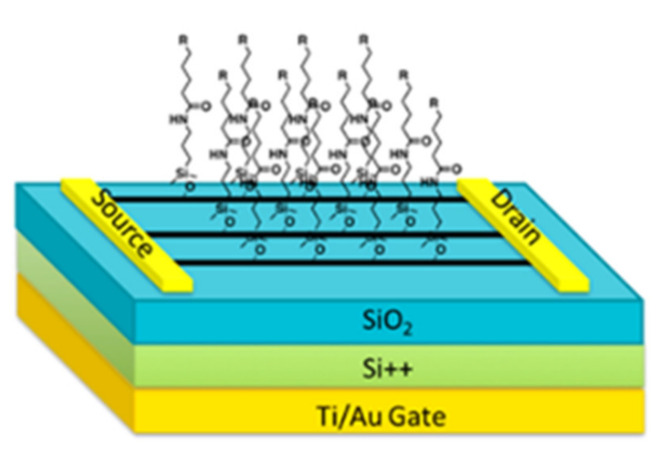
Schematic illustration of device with functionalized SiNWs, where R is: -CH_3_, -C_6_H_5_, -COOH, and -COOCH_3_ [108].

It was shown from Figure 13b that the fluorescence intensities gradually increased with increase of concentration of Cr^3+^ which highlights the sensibility of SiNWs-based fluoroionophores. However, it is well known that rhodamine molecules have non-fluorescence in the spirocyclic form and fluorescence in its open form. The authors explained the fluorescence observed by the formation of complexes rhodamine-Cr^3+^ in solution, that could switch rhodamine molecules to fluorescent open cycle form and enhance the intensities when increasing the concentration of Cr^3+^. The need for gas detection devices to control air pollution in real time continues to grow due to environmental and health concerns. Semiconductor materials such as metals oxides have been intensively studied as conductometric sensors due to their good chemical stability and functional properties [105,106]. In the temperature range from RT to 500 °C, oxygen ionosorbed on n-type semiconductor materials either in molecular (O_2_^−^) or atomic (O^−^) forms, allows to control the conductance properties through the formation of depletion layer. The latter leads to a decrease in conductance of the structure. However, the negatively charged oxygen species can be removed by exposing the semiconductor to a reducing gas and therefore improve its conductance. In short, oxidizing gases reduces the electrical conductance in n-type semiconductor while reducing gases leads to an increase in the conductance. The same trends are observed for p-type gas sensors but in the opposite direction. Note that the target gases can be adsorbed on the surface of a semiconductor either by: physisorption (weak interaction) and chemisorption (strong interaction of gas molecules with solids). In addition, it well established that metal oxide can also act as catalysts for VOCs combustion viz a Mars Van-Krevelen mechanism which involves the consumption and regeneration of the surface lattice and adsorbed oxygen species [107]. Wang et al. explored the effect of the end (functional) group modified SiNWs field effect transistor (FET) upon exposure to various polar and nonpolar volatiles organic compounds (VOCs) [108,109]. Indeed, the functional groups attached to molecular layers can serve as acceptor/donor antenna for signal transduction of VOCs in FET. SiNWs nanowires after an initial pretreatment were dispersed in ethanol and deposited on a substrate formed by p-type silicon wafer (100) with 300 nm thermal oxide having a lower gate Ti/Au (10/200 nm) electrode. The deposition process based on the spray coating approach resulted in the formation of an array of well-aligned of nanowires (1 NW/100 μm^2^). An amine modified device (common backbone) was achieved by immersing the devices in APTES dissolved in dehydrated ethanol before further modifications with the organic groups (Figure 14).

In order to determine the electrical features (current versus voltage), a swept backward between +40 and −40 V was performed under ambient condition afterwards the change in threshold voltage (∆Vth) and in hole mobility (∆µh) were deducted by extrapolation. The bare SiNW showed no clear response to VOCs and/or concentrations while the modified SiNW device exhibited ΔVth sensitivity following the functional groups. An example, In the same conditions, exposure CH_3_-SiNW and COOCH_3_-SiNW to decane led respectively to ΔVth (−1.62 ± 0.28 V) and ΔVth (8.30 ± 0.47 V). The same trend has been observed for hole mobility. The bare SiNW FET showed only weak to negligible Δμh responses except for ethanol which showed a weak positive response. The dependence of the Δµh response turned out to be dependent of the functional groups (CH_3_-SiNW FET > COOCH_3_-SiNW FET > COOH- SiNW FET > C_6_H_5_-SiNW FET) which was assigned to VOC wettability of the molecular layers.

**Figure 15:**
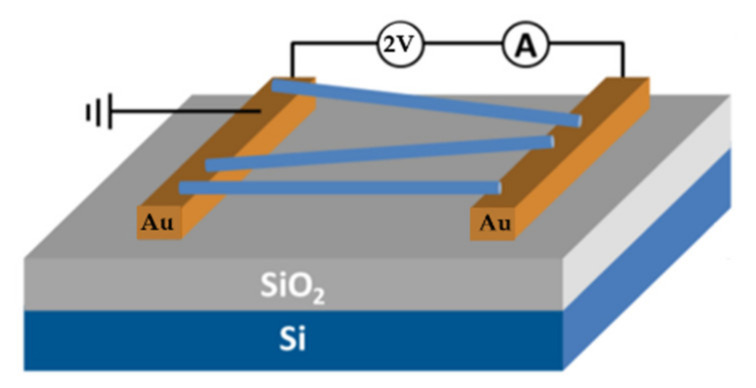
Schematic diagram of the experimental setup.

Hexyltrichlorosilane (HTS) monolayers used to modify SiO_2_/Si NW surfaces in a field effect transistor device also demonstrated to improve the detection of nonpolar VOCs [110]. The detection of VOCs is mainly related to molecular gating. The most important factors underlying are: (i) a change in the dielectric constant and (ii) a change in the charged surface state density of the SiNWs surface. Indeed, the adsorption of VOCs (between or on top monolayer at SiNWs surface) induce a change in its conformation which in turn impact the dielectric constant, the effective dipole moment of the organic monolayer, and/or the charged surface state density of the SiO_2_/monolayer interface. Ultimately, these effects lead to a variation in the conductivity of the SiNWs. Furthermore, Yang et al. designed a room-temperature sensor based on a Tellurium (Te) nanoparticle-modified H-terminated silicon nanowire for ammonia and propylamine detection [111]. The gas sensor was fabricated by coating Te-modified SiNWs directly onto the surface of a ceramic tube having at these ends two Au electrodes and four Pt wires. The sensor was then exposed to the target gas. The characteristics of the gas sensors were studied by measuring the resistance which is expected to increase when it exposed to the target gas. The sensing mechanism was described as follows: the ammonia (or propylamine) gas reduced the oxide layer on Te which leads to reducing the majority carrier density in the Te and thus decreases the conductivity of the sensor [112]. Operating at optimum temperature (35 °C), it was observed a raise of sensor resistance upon increasing concentrations in the detection range of 10–400 ppm for NH_3_ and 5–25 ppm for C_3_H_7_NH_2_. The detection limits/sensitivity responses of sensor were respectively 196 ppb/208% when exposed to 400 ppm ammonia and 174 ppb/164% for 25 ppm propylamine. The sensitivity response, higher than those of Te film-based sensor, [112,113] was attributed to smaller size of nanoparticles since they have a higher activity and thus apt to receive much more oxygen species. Another gas sensor with an equally promising future is undoubtedly the hydrogen sensor. First of all, hydrogen is one of hopeful energy sources owing to its renewability and emits no greenhouse gas [114]. In addition to being flammable and explosive gas at a concentration higher than 4% in air [115], it is a colorless and odorless, which makes it difficult to detect. Hydrogen sensor was developed by dispersing Pd-functionalized SiNWs onto a SiO_2_/Si substrate to form a uniform thin layer. Gold electrodes were subsequently deposited on the sample through a shadow mask and a direct current voltage of 2V was applied (Figure 15) [116].

**Figure 16:**
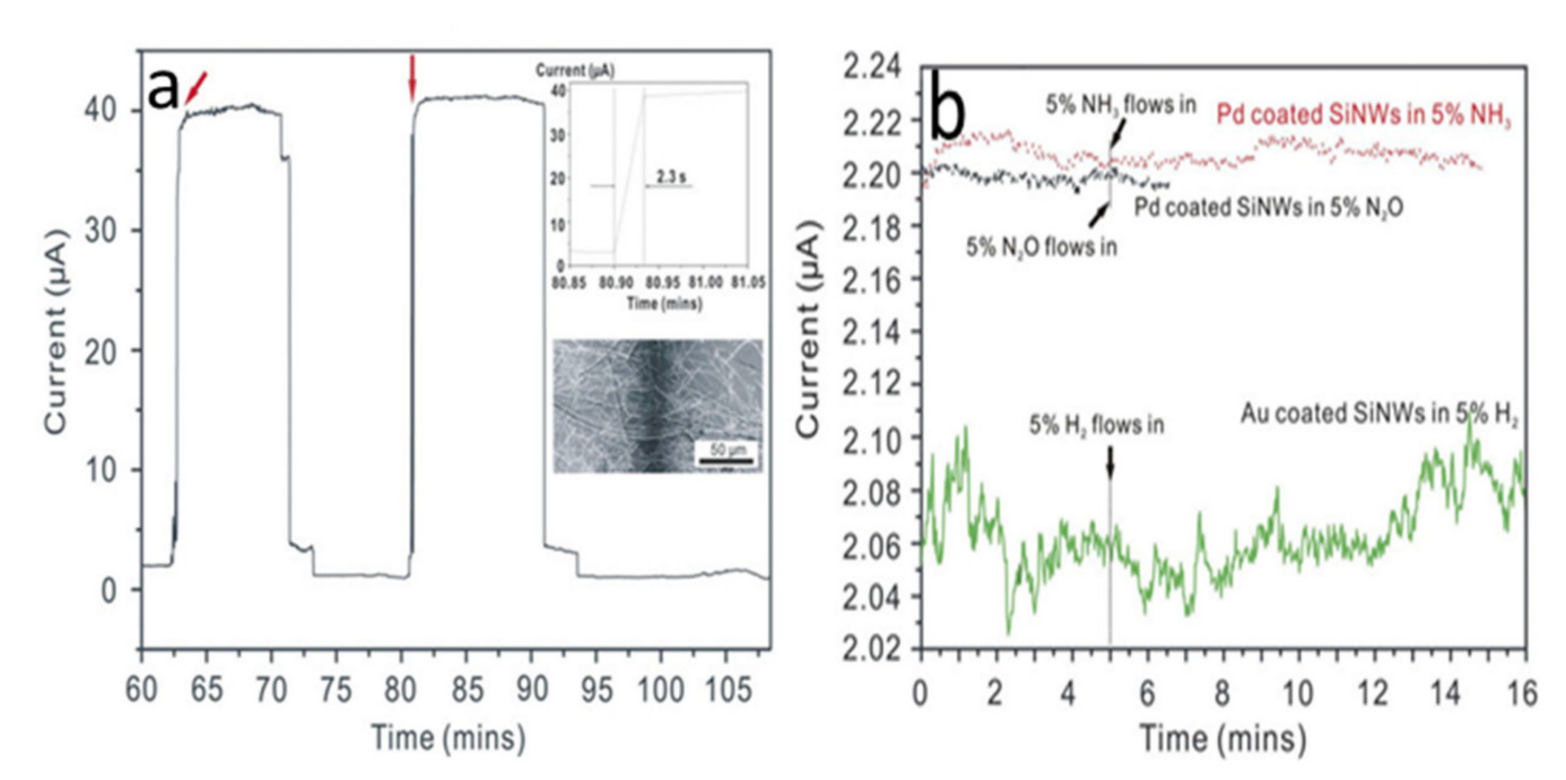
(**a**) Current response of pure H-SiNWs in 5% H_2_ and Pd coated SiNWs in 5% NH_3_ and N_2_O; (**b**) current response of Pd coated SiNWs in 5% H_2_ [117].

Intensity-Potential (I-V) characteristics based on H-terminated SiNWs and Pd-modified SiNWs were measured to evidence hydrogen detection. They observed a small decrease of pure SiNWs conductance after Pd coatings. This was attributed to electron depletion at the Pd and silicon interface. Also, in the presence of 5% H_2_ gas, the current of devices made by dispersion of Pd-SiNWs on SiO_2_/Si increased abruptly from 1.7 to 40 µA and decreased to the background value when the H_2_ supply is stopped (Figure 16a). No response was detected for pure H-SiNWs and no obvious signal was detected when 5% NH_3_ or N_2_O was flowing onto Pd-functionalized SiNWs (Figure 16b). On the other hand, the saturation signal was reached in 2.3 s, much faster than Pd wire [117]. This indicated that Pd-SiNWs is a selective hydrogen sensor with fast response time.

**Figure 17:**
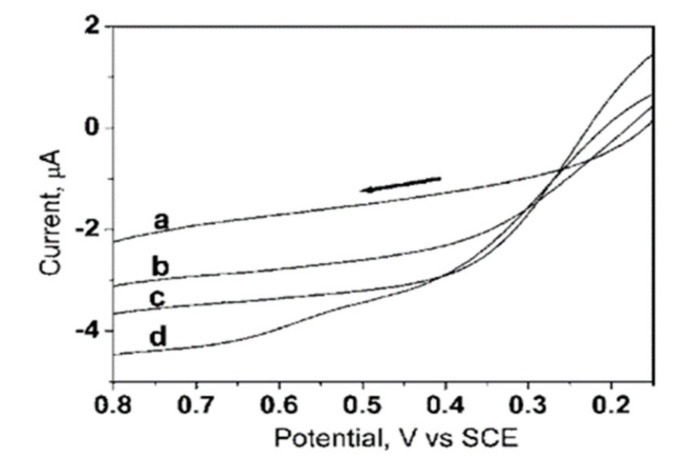
(**a**) Linear sweep voltammograms of biosensors in pH 7.0 phosphate buffer solution without glucose. Amperometric response based on as grown SiNWs (**b**) etched SiNWs (**c**) COOH-SiNWs (**d**) in presence of 2 mM glucose. Scan rate 5 mV/s. [120].

Organophosphonate and thiol-terminated SiNWs served as platforms to attach peptide nucleotide acid (PNA) probes for DNA sensing [118,119]. In the last case, DNA detection was achieved by monitoring the conductance, via current-voltage measurements, of the functionalized SiNWs at various concentration of target DNA in solution. After the injection 10 pM DNA solution over the sensors surface, a significant steady-state conductance increase was observed for all the sensors indicating the hybridization of the target DNA with the probes on the surfaces of the nanowires. Further injections showed only a slightly conductance increased due to the few unoccupied binding sites available. On another note, SiNWs have good biocompatibility with a broad range of enzyme molecules and good electron-transfer mediator for amperometric biosensor. Chen et al. demonstrated that SiNWs surface treatment affects the electron-transfer conductivity throughout the electrocatalytic detection of glucose [120]. They have used three different surfaces treated SiNWs: the as-grown prepared by oxide-assisted growth, those without the surrounding oxide sheath and carboxylic acid-functionalized SiNWs (COOH-SiNWs). A mixture of SiNWs and glucose oxidase dispersed in de-ionized distilled water was coated on Pt electrodes, then their performances were evaluated by electrochemical means. Each of the nanowires exhibited a current increase at +0.2 V in presence of 2 mM glucose solution. However, COOH-SiNW presented the highest current values and a sensitivity three times greater than that of etched and as-grown SiNWs (Figure 17). Several examples can be found in the literature [17,55,78]. Modified silicon nanowires have also been applied for the development of humidity sensors, which are important in the agriculture, industrial production and food storage sectors. Indeed, SiNWs have attracted more attention as humidity sensing materials due to their larger surface-to-volume ratio and their hydrophilic surface favoring water molecules adsorption [121]. Nevertheless, the hydroxyl groups on their surfaces lead to a long response time and obvious hysteresis.

Briefly, high performance humidity sensors are required to have a linear response, high response, fast response time, chemical and physical stability, wide humidity operating range and low cost. Zhang et al. compared the humidity sensor capacity of SiNWs as-synthesized using chemical etching procedure and SiNWs modified with Hexamethyldisilazane (SiNWs-HDMS) [122]. The humidity sensing behavior of SiNWs was evaluated by capacitance measurements under different relative humidity. When the relative humidity changed from 11.3% to 93%, the corresponding capacitance variation (ΔC/C) was respectively 78.7% and 16.4% for SiNWs and SiNWs-HDMS. The reason for the capacitance changes was attributed to the adsorption/desorption process of water molecules on the surface of humidity sensing material. Concretely these results mean that less water molecules were adsorbed onto modified silicon nanowires and are therefore less sensitive than SiNWs as-synthesized. However, after HDMS treatment the maximum hysteresis rate was 1.1% (eight times smaller than that of SiNWs based sensor) and the response time at high relative humidity level was 132 s versus 350 s for unmodified silicon nanowires. HDMS-functionalized SiNWs based humidity sensor exhibited thus a better performance including short response time and lower hysteresis. Apart from organic groups, inorganic compounds were also used to enhance the moisture detection of SiNWs. Taghinejad et al. reported a technique to achieve high-sensitivity humidity sensors based on the post-growth doping of SiNWs with phosphorus [10]. In order to point out the doping effect on moisture detection, capacitive-voltage analysis was performed on three different sensor viz low density, high density and doped- high density SiNWs under relative humidity of 30%. A highest capacitance value was found for doped nanowires due to enhancement of conductivity. Likewise, the relative sensitivity (ΔC/C) of characterized nanowires showed that doping increased the sensitivity which however leads to a longer response time. Given that infrared data revealed that the doped SiNWs were more hydrophobic, the improved sensitivity has been assigned to their conductivities. The same observations were made with Ni/SiNWs nanocomposites using as sensitive materials to design a capacitive humidity sensor [123]. Table 2 summarizes some examples of these reactive surface groupings on SiNWs.

## 4. Concluding Remarks and Outlook

In summary, it should be noted that silicon nanowires, due to their high surface-to-volume ratio, are definitely promising materials for the development of real applications. We have presented various methods, both in solution and in gas phase, that have been developed to deposit organic layers or doping agents on silicon surfaces. On the other hand, the modification of silicon nanowires surface both by covalent bonding or electrostatic interaction leads to a change of surface properties and increases the selectivity. Despite the many functionalization approaches, silanization remains the most applied for its simplicity and the high surface loading. Also, we have reported that there are various surface characterization techniques, such as X-ray photoelectron spectrometry, Transmission electron microscopy, scanning electron microscopy, infrared spectroscopy, or ellipsometry can be used to demonstrate the evidence of characterization. Furthermore, we observed that silicon nanowires functionalized or not found a lot of applications as sensors in several domains like gas, ions or humidity detection as summarized in Table 2. SiNW based radio frequency sensors have been clearly shown to improve moisture detection due to their hydrophilic surface. Although the realization of such sensors is simple, they do not give an absolute value but a threshold value. Nonetheless, the manufacture of sensor devices based on silicon nanowires requires a prior surface functionalization to monitor their physical/chemical properties and thus improve their detection capacity. SiNW-based fluoroionophores favored the increase in fluorescence intensities when adding different concentrations of heavy metals, as they allow high mobility of carriers. Also, the functional groups attached to the oxidized silicon layers can serve as an acceptor/donor antenna for signal transduction of VOCs in the FET. This involves a change in the conformation of the layer which in turn impacts the dielectric constant and therefore the conductance. On the other hand, SiNWs have good biocompatibility with a wide range of enzymatic molecules and consequently can play the role of detection agent in biological sensors. Carboxylic acid functionalized SiNWs exhibited a high current in glucose detection compared to unmodified silicon nanowires. Finally, besides the sensitivity and selectivity, the speed of response is an important factor in sensor development. HMDS-modified SiNWs based humidity sensor exhibited the improved humidity sensing behavior, such as linearity, or response time compared to the SiNWs based sensor. Despite the advantages of functionalized silicon nanowires and the wide range of sensing applications, the major drawback remains the low long-term stability of the organic layers attached to the nanowires under harsh temperature and environmental conditions. The recent developments in the sensor field report a future generation of sensor devices that are chipless RFID. Taking into account the intrinsic properties of silicon nanowires and the advances in technology, we could imagine the design of RFID sensors based on modified silicon nanowires. The functionalization of an appropriate detection agent on their surfaces could lead to applications like RFID humidity, gas, or temperature sensors which would be really practical in our daily life.

## Figures and Tables

**Table 1:** Comparison of the different functionalization methods.

Functionalization Method	Simplicity of Implementation	Sensor Designing	Advantages/Disadvantages	Process Temperature (°C)	Reference
Hydrosilylation	Versatile approach	Well adapted for large scale sensor development	Improve the sensing effect; High quality films obtained; Large excess of unsaturated molecules required	100–230	[17,46,48,52]
Silanization	Easy to implement	Most applied for sensor design	High surface loading; Plausible condensation of organosilanes on the surface	25–130	[68,76,84,91]
Esterification	Stepwise functionalization	Little used technique	Lower hydrolytic stability; Eco-friendly process	25	[24]
Phosphorization	difficult to prepare well-defined surface for functionalization	ill-adapted for industrial applications	High surface coverage; Excellent thermal stability; Stable P-O-C bonds upon hydrolysis. Unsuitable for attaching organic groups; Long processing time (48h)	140	[25,28,32,35]

**Table 2:** Some examples of organosilane/organophosponate used to modify silicon nanowire.

Sensor Type	Surface Modification	Doping Agent	Receptor	Sensing	Type of Measurement	Reference
Gas	APTES	-	CH_3_, C_6_H_5_, COOH, COOCH_3_	Volatile organic compounds, TNT, Ammonia, Propylamine	In touch	[104,105,106,107,108,109,112,124]
	HTS	-	-			
Humidity	HDMS	-	-	Water	In touch	[18,113]
	-	POCl_3_	-			
Hydrogen	-	Pd	-		In touch	[115]
Ions	Fluoroionophores	-	-	Pb^2+^, Cr^3+^, Cd^2+^, Hg^2+^	remote	[104]
Biological	11-hydroxyundecylphosphonate	-	PNA	DNA		[110,111,112]
	MPTMS	-	PNA	DNA	In touch	
	10-undecylenic acid (UDA)	-	-	Glucose		
pH	Polyaniline	-	DNA	H^+^	In touch	[125]

## Data Availability

Not applicable.
